# Hospital-acquired listeriosis linked to a persistently contaminated milkshake machine

**DOI:** 10.1017/S0950268816003198

**Published:** 2017-01-09

**Authors:** E. MAZENGIA, V. KAWAKAMI, K. RIETBERG, M. KAY, P. WYMAN, C. SKILTON, A. ABERRA, J. BOONYARATANAKORNKIT, A. P. LIMAYE, S. A. PERGAM, E. WHIMBEY, R. J. OLSEN-SCRIBNER, J. S. DUCHIN

**Affiliations:** 1 Food Protection Program of Environmental Health Division, Public Health – Seattle & King County, Seattle, WA, USA; 2 Communicable Disease Epidemiology and Immunization Section, Public Health – Seattle & King County, Seattle, WA, USA; 3 Epidemic Intelligence Service, Division of Scientific Education and Professional Development, Centers for Disease Control and Prevention, Atlanta, GA, USA; 4 University of Washington, Seattle, WA, USA; 5 Fred Hutchinson Cancer Research Center, Seattle, WA, USA

**Keywords:** Hospital-acquired (nosocomial) infections, *Listeria*, listeriosis, milkshake machine

## Abstract

One case of hospital-acquired listeriosis was linked to milkshakes produced in a commercial-grade shake freezer machine. This machine was found to be contaminated with a strain of *Listeria monocytogenes* epidemiologically and molecularly linked to a contaminated pasteurized, dairy-based ice cream product at the same hospital a year earlier, despite repeated cleaning and sanitizing. Healthcare facilities should be aware of the potential for prolonged *Listeria* contamination of food service equipment. In addition, healthcare providers should consider counselling persons who have an increased risk for *Listeria* infections regarding foods that have caused *Listeria* infections. The prevalence of persistent *Listeria* contamination of commercial-grade milkshake machines in healthcare facilities and the risk associated with serving dairy-based ice cream products to hospitalized patients at increased risk for invasive *L. monocytogenes* infections should be further evaluated.

*Listeria monocytogenes* is a foodborne bacterial pathogen known to cause invasive infections disproportionally in immunocompromised persons, adults aged ⩾65 years, and newborns; it can also result in spontaneous abortions, stillbirth, and fetal death in pregnant women [1–3]. Annually, in the United States, *Listeria* infections cause an estimated 1460 hospitalizations and 260 deaths [2].

Milkshakes are a high-protein, caloric-dense, pasteurized dairy-based ice cream product served to hospital patients regularly and are also widely available in the community. In recent years, there have been two *Listeria* outbreaks in the United States linked to the consumption of pasteurized dairy-based ice cream products [4, 5]. Currently, there are no recommendations by either FDA or CDC to limit or restrict the consumption of pasteurized ice cream products by high-risk populations for invasive listeriosis [3, 6, 7].

Shake freezers are commercial-grade machines that hold dairy-based ice cream mix at a safe holding temperature (<5 °C) while allowing quick preparation of a variety of frozen beverages including milkshakes. The main components of shake freezers are a holding mix tank, mix feed system, freezing cylinder, beater, and dispensing nozzle. The refrigerated holding mix tank (hopper) holds and refrigerates the liquid ice-cream mix product until it is ready to be injected into the freezing cylinder through the mix feed system. Once in the freezing cylinder, the ice-cream mix freezes to the walls of the cylinder and the beater scrapes the mix off the cylinder walls until it achieves the proper consistency for the product to be served through the dispensing nozzle.

On 25 November 2015, Washington State Department of Health (WA DOH) notified Public Health – Seattle & King County (PHSKC) of a hospitalized patient with a *L. monocytogenes* blood culture isolate that had a rare pulsed-field gel electrophoresis (PFGE) pattern closely related to two hospital-acquired *L. monocytogenes*-infected patients that were hospitalized at the same King County, Washington State hospital (hospital A) 1 year earlier. A PHSKC investigation of the initial 2014 outbreak determined that a locally produced, commercial pasteurized ice cream mix was the source of the *L. monocytogenes* outbreak [5]. The 2014 outbreak resulted in the recall of all products produced by the ice cream manufacturer, temporary closure of the manufacturer, and suspension of the shake freezer machine use at the hospital [5]. Hospital A had two commercial-grade shake freezer machines of the same model that were used to produce milkshakes; one was in the hospital cafeteria (machine A) and the other was used exclusively for hospitalized patients (machine B). Hospital A shake freezers had two separate 18·9-litre refrigerated mix hoppers and 6·6-litre freezing cylinders, which were used to serve both vanilla and chocolate milkshakes.

To prevent future *L. monocytogenes* infections, hospital personnel were instructed to disassemble, thoroughly clean and sanitize all contact surfaces of both machines as outlined in the manufacturer's operating manual [8]. In brief, hospital A personnel emptied the machines of all ice cream product, flushed with water and a manufacturer-approved, powdered chlorinated detergent sanitizer solution was used to sanitize the interior of the machines. The machines were disassembled to brush clean, sanitize and air-dry the internal components coming into direct contact with ice cream mix product before reassembling. The cleaning and sanitizing process took hospital A personnel more than 2 h for each machine. On 30 December 2014, hospital A personnel cleaned and sanitized both machines and collected environmental samples for *L. monocytogenes* testing at a private food laboratory (laboratory A) using two methods; sponge swabs and water rinse samples. Sponge swabs were collected from the hopper and exterior surfaces of the dispensing nozzles while water rinse samples consisted of sterile water rinsed through the machine and collected from the dispensing nozzle. A total of eight samples were collected (two rinse and two swab samples for each machine); both rinse samples from machine B were positive for *L. monocytogenes*. Subsequently, hospital A personnel repeated cleaning and sanitation of both machines several times; on 5 January 2015, repeat testing using the same environmental sample collection methods resulted in both machines testing negative for *L. monocytogenes* (Fig. 1). Hospital A resumed operation of both machines using ice cream mix product from a different source and increased machine cleaning and sanitizing from weekly to twice weekly. In addition, hospital A implemented a policy of not using leftover milkshake product and replacing rubber gaskets every 3 months.

**Fig. 1.**
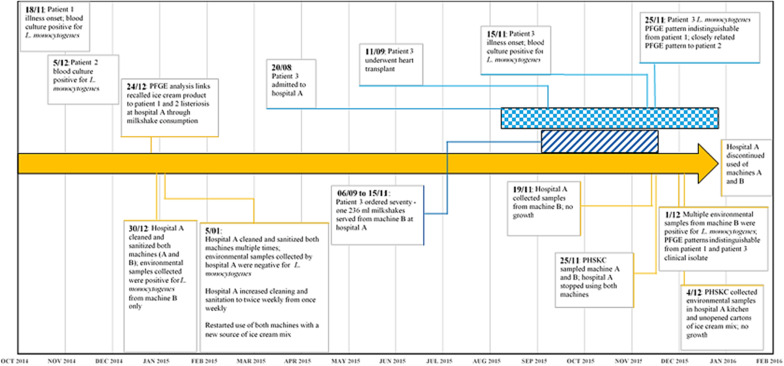
Timeline of epidemiological, environmental and laboratory investigation of a hospital-acquired listeriosis linked to milkshakes produced in a persistently contaminated shake freezer machine. 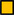, Machines A and B at hospital A; 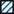, patient 3's milkshake consumption at hospital A; 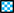, patient 3's hospitalization at hospital A.

The two patients in the 2014 *L. monocytogenes* outbreak were non-Hispanic men in their fifties who were hospitalized at hospital A. Patient 1, was admitted to hospital A on 2 November 2014, underwent heart transplantation on 3 November 2014 and developed fever and chills on 18 November 2014, with subsequent blood cultures positive for *L. monocytogenes* [5]. Patient 2 was admitted to hospital A on 27 October 2014, for a gastrointestinal bleed complicated by enterococcal bacteraemia; within 24 h of discharge on 4 December 2014, he developed fever, fatigue, headache and confusion with subsequent blood cultures positive for *L. monocytogenes* [5]. Both patients were interviewed with the CDC's standardized *Listeria* Initiative questionnaire [9] and detailed hospital dietary records indicated that while hospitalized, patient 1 ordered milkshakes 2–4 times per week and patient 2 ordered two milkshakes per day which were all produced in machine B [5].

Patient 3 was a woman in her forties admitted to hospital A on 20 August 2015, who subsequently underwent a heart transplantation on 11 September 2015. Two separate sets of blood cultures obtained after she became febrile on hospitalization day 85 (15 November 2015) tested positive for *L. monocytogenes*. Patient 3 received intravenous ampicillin and recovered without complications.

*L. monocytogenes* is an uncommon recipient-derived pathogen in solid organ transplant patients [10]. However, the United Network for Organ Sharing confirmed to hospital A that other solid organ recipients who shared the same donor as patient 3 did not have clinical or laboratory evidence of an *L. monocytogenes* infection.

Patient 3 was interviewed with the CDC's standardized *Listeria* Initiative questionnaire [9] and her detailed hospital dietary records were reviewed. Based on patient 3′s interview and dietary records, patient 3 ordered 71 236 ml milkshakes served from hospital A's machine B (range 0–3 servings/day) during her exposure period, defined as the 70 days before symptom onset.

On 19 November 2015, hospital A collected 15 samples from machine B for *Listeria* testing at laboratory A. The sampling method was similar to previous methods used by hospital A; cleaning and sanitizing the machine prior to collecting eight sponge swab samples (hoppers and exterior surfaces of the dispensing nozzles) and seven rinse samples (sterile water was placed in the hopper and passed through the system and collected upon exit from the dispensing nozzles). On 23 November 2015, all 15 samples tested negative for *Listeria* species, and hospital A resumed use of machine B to serve patients milkshakes.

On 25 November 2015, the clinical isolate for patient 3 yielded an indistinguishable PFGE pattern (pattern A) from the 2014 outbreak patient 1 and was closely related to 2014 outbreak patient 3, differing by two bands (pattern B). PHSKC instructed hospital A to stop serving milkshakes from both shake freezer machines.

Sampling for *L. monocytogenes* was conducted by PHSKC during an environmental inspection of hospital A's kitchen on 25 November 2015; Eighteen samples were collected from machine B which included dispensed milkshake product, ice cream mix scooped directly from the hopper, and sponge swabs of the freezer cylinder, an internal component that required some disassembly to access (Table 1). At the time of sample collection, machine A was not in use so only eight sponge swab samples were collected. In addition, two unopened ice cream mix 1·89-litre cartons from different lots obtained from hospital A walk-in coolers were submitted for *Listeria* testing. On 4 December 2015, PHSKC collected an additional nine unopened ice cream mix 1·89-litre cartons from different lots (total of six lots) and 25 additional sponge swab samples of surfaces throughout the hospital kitchen to assess the potential for persistent environmental *Listeria* contamination of other food service areas.

**Table 1.** Sampling for *Listeria monocytogenes* at hospital A, November to December, 2015

Collection date	Sample location	Machine	No. of samples	Collection method*	*L. monocytogenes* culture results
25 Nov. 2015	Dispensed mix	B	6	Dispensed into Whirlpak	Positive (*n* = 3)
25 Nov. 2015	Mix tank (hopper)	B	2	Ice cream mix scooped from reservoir	Positive (*n* = 1)
25 Nov. 2015	Mix tank (hopper)	B	4	Sponge swab of side walls	Positive (*n* = 2)
25 Nov. 2015	Freezer cylinder	B	6	Sponge swab of nozzle pieces	Positive (*n* = 3)
25 Nov. 2015	Mix tank (hopper)	A	2	Sponge swab of side walls	Negative
25 Nov. 2015	Freezer cylinder	A	6	Sponge swab of nozzle pieces	Negative
25 Nov. 2015	Liquid ice cream mix	n.a.	2†	Unopened 1·89-l cartons	Negative
4 Dec. 2015	Liquid ice cream mix	n.a.	9†	Unopened 1·89-l cartons	Negative
4 Dec. 2015	Kitchen surfaces‡	n.a.	25	Sponge swabs of various kitchen surfaces	Negative

n.a., Not applicable.

* All samples were collected by PHSKC Food Protection Program personnel.

† Unopened ice cream mix cartons were from different lots (*n* = 6) collected from hospital A walk-in cooler.

‡ Hospital A kitchen surfaces sampled included doorknobs, drawer shelves, food preparation tables, room service carts, sinks, floors, and floor drains.

Testing of food and environmental samples was performed at the same private food laboratory (laboratory A) previously used by hospital A. Samples were enriched in a proprietary liquid culture medium (IEH M1 Medium with Gram-positive additive and nalidixic acid) for 24 h at 35 °C. Enriched samples were initially analysed by polymerase chain reaction (PCR); *Listeria* spp. samples that were ‘Initial Reactive’ after completion of the primary PCR were concentrated using a *Listeria*-specific immunomagnetic separation procedure followed by a secondary PCR for *Listeria*. Samples that were presumptive positive after the secondary PCR were confirmed using culture procedures as described in the FDA Bacteriological Analytical Manual [11]. Confirmatory testing of clinical isolates from patients was performed at WA DOH Public Health Laboratories (PHL) utilizing traditional culture-based and biochemical identification methods for *L. monocytogenes* confirmation. Two-enzyme (*Asc*I and *Apa*I) PFGE analysis was performed at laboratory A and WA DOH PHL using methods described previously [12, 13].

Six of 20 sponge swab machine samples and 3/6 dispensed milkshake products tested positive for *L. monocytogenes*. All nine positive samples which were indistinguishable by PFGE pattern (pattern A) from clinical isolates of patients 1 and 3, were from machine B. A retrospective review of the national CDC PulseNet PFGE database indicated pattern A was only previously detected in the 2014 *L. monocytogenes* outbreak at hospital A. Machine A environmental samples for *L. monocytogenes* were negative. Similarly, unopened milkshake dairy mix product samples and hospital A cafeteria surfaces were negative for *L. monocytogenes.*

PHSKC investigated one case of hospital-acquired listeriosis in which the epidemiological and laboratory evidence indicated that the source of infection was a commercial-grade freezer shake machine persistently contaminated with *L. monocytogenes* for at least 1 year, despite twice-weekly cleaning and sanitizing. The contamination of this equipment went undetected despite two rounds of environmental sampling. Reintroduction of the rare 2014 *L. monocytogenes* outbreak strain to hospital A is unlikely because follow-up environmental cultures of the food preparation and cleaning areas after patient 3′s *L. monocytogenes* exposure period were negative. In addition, the hospital had changed its source of ice cream product mix before identification of patient 3′s *L. monocytogenes* exposure period and 11 unopened cartons from different lots of the new ice cream product mix tested negative for *L. monocytogenes*. This outbreak underscores the challenges of cleaning and sanitizing inaccessible food contact surfaces, and the opportunity for *L. monocytogenes* to persist in food service equipment and cause human infections for extended periods.

*L. monocytogenes* has been known to persist in food-processing plants for years, particularly on equipment surfaces that cannot be reached by sanitizers (i.e. niches), which contributes to the foodborne transmission of *L. monocytogenes* [14, 15]. Further, *L. monocytogenes* is well adapted to cold temperatures, growing and surviving at refrigeration temperatures ranging from –0·5 to 9·3 °C [16]. *L. monocytogenes* is also known to rapidly attach to different surfaces used in food processing and facilities, forming either strong cell adsorption or biofilm [14]. Although *L. monocytogenes*’ ability to survive in different conditions contributes to its persistence, current knowledge indicates that niches are the main factor that allows *L. monocytogenes* to persist in the environment [14].

Hospital food service operations should have a *L. monocytogenes* control plan that addresses kitchen equipment design, adherence to approved cleaning and sanitation schedules, and ongoing stringent sampling of suspect products and equipment [15, 17]. Numerous commercial milkshake machines exist on the market. Certain models use self-contained mix products in disposable bags where food products do not come in contact with reusable parts; others, including those used by hospital A, have reservoirs, pipes, and mixers that make contact with mix products. *Listeria*’s ability to form strong attachments to food contact surfaces, along with the high fat content of the dairy-based ice cream mix, might create an ideal environment for *L. monocytogenes* to resist cleaning, thereby contributing to its persistence in both accessible and inaccessible machine parts. The operating manual for the machines used by hospital A recommends cleaning and sanitizing these machines daily, although hospital A reports that the equipment representative recommended weekly cleaning. Hospital A chose to clean and sanitize twice weekly, which took more than 2 h per machine. Because of associated cleaning challenges, hospital A permanently discontinued machine use during the investigation of patient 3's listeriosis. To minimize risk of bacterium persistence during operational periods, adherence to manufacturers’ cleaning and sanitizing recommendations should be practised. However, for both commercial and healthcare facilities, it is unknown to what extent operators conform to these guidelines. To minimize risk of introduction of *L. monocytogenes* into the machine, facilities serving patients at high risk for listeriosis should also consider restricting use of equipment that permits direct contact of food product and machine surfaces.

During the study period, three sets of environmental sponge swab samples from both machines that had been fully cleaned and sanitized tested negative for *L. monocytogenes*, but multiple sponge swab samples of one machine's surfaces with milkshake product still inside tested positive. Testing after cleaning and sanitizing minimizes the likelihood of recovering the bacterium; sampling plans should consider testing equipment before cleaning and during use to maximize the likelihood of recovering *Listeria* species and other pathogens. Further, robust sampling techniques (e.g. sponge swab sampling of hard-to-clean environmental niches), should be considered.

Our investigation is subject to two limitations. Although unlikely, we did not investigate if the producer of the ice cream mix served to patient 3 had used any of the same manufacturer's facilities, equipment, or ingredients as the ice cream mix producer implicated in the 2014 *L. monocytogenes* outbreak. The second limitation is because the *Listeria* testing of the environmental samples was qualitative (presence-absence) and not quantitative, we were unable to determine the extent of *L. monocytogenes* contamination in different components of the machines.

Healthcare facilities’ re-evaluation of their current restricted food polices to include elimination of dairy-based ice cream products for immunocompromised, hospitalized patients could help prevent hospital-acquired foodborne *Listeria* infections. A discussion of restricted food policy should include investigating alternate milkshake products that have minimal storage and preparation requirements, which could minimize post-pasteurization *Listeria* contamination. The prevalence of persistent *Listeria* contamination of shake freezer in healthcare facilities and the risk associated with serving pasteurized dairy-based milkshakes to hospitalized patients at increased risk for invasive *L. monocytogenes* infections should be further evaluated.
